# Biomarker-based prognostic stratification of young adult glioblastoma

**DOI:** 10.18632/oncotarget.5456

**Published:** 2015-10-05

**Authors:** Rui-qi Zhang, Zhifeng Shi, Hong Chen, Nellie Yuk-Fei Chung, Zi Yin, Kay Ka-Wai Li, Danny Tat-Ming Chan, Wai Sang Poon, Jinsong Wu, Liangfu Zhou, Aden Ka-yin Chan, Ying Mao, Ho-Keung Ng

**Affiliations:** ^1^ Department of Anatomical and Cellular Pathology, Chinese University of Hong Kong, Hong Kong, China; ^2^ Shenzhen Research Institute, Chinese University of Hong Kong, Hong Kong, China; ^3^ Neurosurgery Division, Department of Surgery, Chinese University of Hong Kong, Hong Kong, China; ^4^ Department of Neurosurgery, Huashan Hospital, Fudan University, Shanghai, China; ^5^ Department of Neuropathology, Huashan Hospital, Fudan University, Shanghai, China

**Keywords:** glioblastoma, IDH1, BRAF, H3F3A, prognostication

## Abstract

While the predominant elderly and the pediatric glioblastomas have been extensively investigated, young adult glioblastomas were understudied. In this study, we sought to stratify young adult glioblastomas by *BRAF*, *H3F3A* and *IDH1* mutations and examine the clinical relevance of the biomarkers. In 107 glioblastomas aged from 17 to 35 years, mutually exclusive *BRAF*-V600E (15%), *H3F3A*-K27M (15.9%), *H3F3A*-G34R/V (2.8%) and *IDH1*-R132H (16.8%) mutations were identified in over half of the cases. *EGFR* amplification and *TERT*p mutation were only detected in 3.7% and 8.4% in young adult glioblastomas, respectively. *BRAF*-V600E identified a clinically favorable subset of glioblastomas with younger age, frequent *CDKN2A* homozygous deletion, and was more amendable to surgical resection. *H3F3A*-K27M mutated glioblastomas were tightly associated with midline locations and showed dismal prognosis. *IDH1*-R132H was associated with older age and favorable outcome. Interestingly, tumors with positive PDGFRA immunohistochemical expression exhibited poorer prognosis and identified an aggressive subset of tumors among K27M mutated glioblastomas. Combining *BRAF*, *H3F3A* and *IDH1* mutations allowed stratification of young adult glioblastomas into four prognostic subgroups. In summary, our study demonstrates the clinical values of stratifying young adult glioblastomas with *BRAF*, *H3F3A* and *IDH1* mutations, which has important implications in refining prognostic classification of glioblastomas.

## INTRODUCTION

Glioblastoma is the commonest and most devastating primary brain cancer [1]. The disease has a universally fatal prognosis despite aggressive treatment in which over 85% of patients die within two years [2]. While the median age group is middle age to elderly, a smaller numbers of cases are found in young adults and children [2, 3]. The predominant group of middle aged and elderly glioblastomas has been extensively investigated. Molecular classification in glioblastomas based on gene expression profiles classified the heterogeneous disease into proneural, neural, classical and mesenchymal subtypes, with each subtype carried distinct genomic aberrations [4]. *Isocitrate dehydrogenase-1 (IDH1)* mutation or *platelet-derived growth factor receptor alpha* (*PDGFRA*) amplification, *epidermal growth factor receptor* (*EGFR*) amplification and *neurofibromin 1* (*NF1*) mutations were associated with proneural, classical and mesenchymal glioblastomas, respectively [4]. Genome-wide methylation study further identified a subset of adult glioblastoma with glioma-CpG island methylation phenotype (G-CIMP) which was enriched in proneural subgroup, tightly associated with *IDH1* mutation and exhibiting favorable prognosis [5]. For the younger group, only the children's glioblastomas has been extensively studied [6–9]. Distinct genomic aberrations including *PDGFRA* alterations and hotspot mutations in *histone 3.3* (*H3F3A*) at codons 27 (K27) and 34 (G34) as well as *histone 3.1* (*HIST1H3B*) at codon 27 (K27) were frequently found in glioblastomas in children [6–8]. Genomic study in combined series of pediatric and adult glioblastomas further identified age-specific biological subgroups which can be defined by driver events including *H3F3A*-K27M, *H3F3A*-G34R/V and *IDH1* mutations, strongly indicating that glioblastomas are different diseases in different age groups [10, 11]. Recent study also reported the activating mutation *BRAF*-V600E identified a distinct clinical subgroup of pediatric high grade gliomas [12]. While current literature has been only focused in glioblastomas of either children or older patients and in particular, vast majority of adult glioblastomas studied were above 35 years as a result of the skewed distribution towards older age (median age 64 years) [2], the young adult age group was known to have better prognosis [3, 13] but was understudied in the literature. In this regards, we investigate a set of subgroup-defining molecular biomarkers in young adult glioblastomas aged from 17 to 35 years and evaluate the prognostic impact of the biomarkers. Our study reveals that *BRAF*, *H3F3A* and *IDH1* mutations are associated with distinct clinical features and can stratify young adult glioblastomas into prognostic subgroups, which have important clinical implications in refining the prognostic classification of glioblastomas in young adults.

## RESULTS

### Cohort characteristics

**Figure 1:**
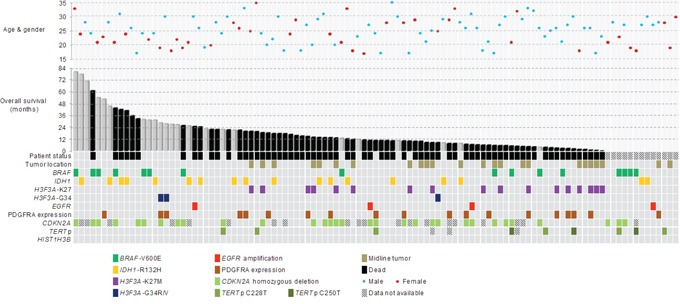
Clinical, genetic and molecular characteristics of 107 young adult glioblastomas aging from 17 years to 35 years Mutation rates of *BRAF*, *H3F3A* and *IDH1* were 15% (16/107), 18.7% (20/107) and 16.8% (18/107), respectively. No *HIST1H3B* mutation was detected. *BRAF*-V600E mutation was associated with *CDKN2A* deletion (*p* = 0.0002) and younger age (*p* = 0.013). *H3F3A*-K27M mutation was associated with midline tumor location (*p* < 0.00001). Positive PDGFRA expression co-occurred in 50% of *H3F3A* mutated tumors. *IDH1*-R132H mutation was associated with older age (*p* = 0.012). *BRAF*, *IDH1*, *H3F3A*-G34R/V mutations and *EGFR* amplification predominantly developed in hemispheric locations.

Clinical and molecular data of the cohort was summarized in Table 1 and Figure 1. The age of the young adult glioblastoma cohort ranged from 17 years to 35 years. The mean and median ages were 25 years, respectively. The male-to-female ratio was 1:1.61. Eighty-eight tumors (82.2%) were located in cerebral hemisphere. There were 51 tumors (47.7%) involving frontal lobe, 18 tumors (16.8%) involving parietal lobe, 30 tumors (28%) involving temporal lobe and seven tumors (6.5%) involving occipital lobe, with 18 cases (16.8%) affected two cerebral lobes and five frontal tumors also affected corpus callosum (four cases) and lateral ventricle (one case). Twenty-four tumors (22.4%) affected midline structures including one case (0.9%) in basal ganglia, seven cases (6.5%) in thalamus, six cases (5.6%) in ventricular system, five cases (4.7%) in corpus callosum, one case (0.9%) in cerebellum and four cases (3.7%) in spinal cord. Treatment data in operation and chemo-radiotherapy was available in 87 patients (81.3%) and 80 patients (74.8%), respectively. Fifty-eight of 87 patients (67%) received total resection. Sixty-one of 80 patients (76.3%) received radiotherapy and 64 of 80 patients (80%) received chemotherapy, with 52 of 80 patients (65%) received concomitant chemo-radiotherapy. Survival data was available in 94 patients (87.9%). The median overall survival and median follow-up were 14.7 months and 31.6 months, respectively.

**Table 1:** Clinical and molecular data of the young adult glioblastoma cohort

	*n* = 107
Gender (Male/Female)	66/41
Age (Mean/median/range)	25.0/25/(17–35)
Tumor location	
Cerebral hemisphere	65 (60.7%)
Cerebellum	1 (0.9%)
Midline structures	18 (16.8%)
More than one location affected	23 (21.5%)
Operation	
Total resection	58 (54.2%)
Non-total resection	29 (27.1%)
Not available	20 (18.7%)
Adjuvant therapy	
Radiotherapy + chemotherapy	52 (48.6%)
Radiotherapy only	9 (8.4%)
Chemotherapy only	12 (11.2%)
No adjuvant therapy	7 (6.5%)
Not available	27 (25.2%)
*BRAF*	
Mutant	16 (15%)
Wild-type	91 (85%)
*IDH1*	
Mutant	18 (16.8%)
Wild-type	89 (83.2%)
*H3F3A*	
K27M	17 (15.9%)
G34R/V	3 (2.8%)
wild-type	87 (81.3%)
*HIST1H3B*	
Mutant	0 (0%)
wild-type	107 (100%)
*EGFR*	
Amplified	4 (3.7%)
Non-amplified	103 (96.3%)
*TERT*p	
C228T mutant	6 (5.6%)
C250T mutant	3 (2.8%)
Wild-type	95 (88.8%)
Not available	3 (2.8%)
*CDKN2A* homozygous deletion	
Yes	31 (29%)
No	56 (52.3%)
Not available	20 (18.7%)
PDGFRA expression	
Positive	33 (30.8%)
Negative	74 (69.2%)

### Young adult glioblastomas show recurrent *BRAF*, *H3F3A* and *IDH1* mutations but infrequent *TERT*p mutation and *EGFR* amplification

Mutational status of *BRAF*, *H3F3A*, *HIST1H3B* and *IDH1* were examined in all 107 young adult glioblastomas. Over half of the cases exhibited mutations in *BRAF*, *IDH* or *H3F3A*. Mutation rates of *BRAF*, *H3F3A* and *IDH1* across the cohort were 15% (16/107), 18.7% (20/107) and 16.8% (18/107), respectively. None of the tumors showed mutation in *HIST1H3B*. Among the *H3F3A* mutated tumors, 17 cases harbored K27M mutation, two cases harbored G34R mutation and one case harbored G34V mutation. All *IDH1* mutated tumors showed *IDH1*-R132H mutation and all *BRAF* mutated tumors showed *BRAF*-V600E mutation. *TERT*p mutation was only identified in 8.7% (9/104) of young adult glioblastomas, including six cases of C228T mutation and three cases of C250T mutation. *EGFR* amplification was detectable in only 3.7% (4/107) of young adult glioblastomas (Table 1 and Supplementary Figure S1). Notably, with the exception of three *BRAF* mutated glioblastomas concurrently harboring *TERT*p mutation, *BRAF*, *H3F3A*, *IDH1*, *TERT*p mutations and *EGFR* amplification were mutually exclusive (Figure 1).

### *BRAF* mutated glioblastomas show frequent *CDKN2A* homozygous deletion and younger patient age

**Figure 2:**
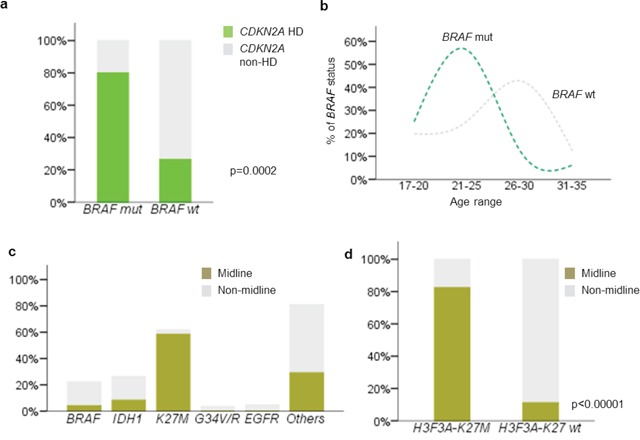
Correlation between clinicopathological and molecular variables of young adult glioblastoma **a.** 80% of *BRAF*-V600E mutated glioblastomas showed concurrent *CDKN2A* deletion. *BRAF*-V600E mutation was closely associated with *CDKN2A* deletion in young adult glioblastoma (*p* = 0.0002). **b.** Patients with mutant *BRAF*-V600E glioblastomas are older than those wild type tumors (*p* = 0.013). **c.** *H3F3A*-K27M mutation was closely associated with midline structures (*p* < 0.00001), while the other mutations, *IDH1*-R132H, *BRAF*-V600E, *H3F3A*-G34R/V and *EGFR* amplification were mainly identified in tumors originated from hemispheric locations. **d.** Only 3.6% of hemispheric glioblastomas harbored *H3F3A*-K27M mutation (*p* < 0.00001). Mut, mutated; wt, wild type; hd, homozygous deletion.

*CDKN2A* homozygous deletion was detected in 35.6% (31/87) of cases with analyzable data (Table 1 and Supplementary Figure S1). Data was non-analyzable in 20 cases due to either weak hybridization signals or strong background fluorescence. Correlating with other molecular biomarkers examined, *CDKN2A* homozygous deletion was associated with *BRAF* mutation. Eighty percent (12/15) of *BRAF* mutated glioblastomas concurrently harbored *CDKN2A* homozygous deletion, compared with 26.4% (19/72) of *BRAF* wild-type glioblastomas (*p* = 0.0002) (Figure 2a). Comparing the age between *BRAF* mutated and *BRAF* wild-type glioblastomas, patients with mutated *BRAF* were younger than those with wild-type. The mean ages of *BRAF* mutated glioblastomas and *BRAF* wild-type glioblastomas were 22.3 years and 25.5 years, respectively (*p* = 0.013) (Figure 2b). Further comparison of patient age between the mutually exclusive molecular subgroups also revealed that *BRAF* mutated glioblastomas were younger than *IDH1* mutated glioblastomas (mean age 22.3 years vs 27.9) (*p* = 0.0008, One-way ANOVA).

### Tumor location differ between *H3F3A*-K27M mutated glioblastomas and other glioblastoma subgroups

Distribution of tumor location according to molecular biomarkers was shown in Figure 2c. *H3F3A*-K27 mutation was found in 58.3% (14/24) of glioblastomas affecting midline structures, including six thalamic tumors, four ventricular tumors and four cervical/thoracic spinal cord tumors. In contrast, only 3.6% (3/83) of hemispheric glioblastomas harbored the mutation (*p* < 0.00001) (Figure 2d). These three K27M mutated hemispheric glioblastomas were all located in temporal/frontal lobe. Young adult glioblastomas with *BRAF*, *IDH1*, *H3F3A*-G34R/V mutations and *EGFR* amplification predominantly developed in hemispheric locations without affecting midline structure, with the exception of one *BRAF* mutated glioblastoma in corpus callosum and three *IDH1* mutated glioblastomas in basal ganglia (one case) and corpus callosum (two cases).

### Frequent PDGFRA expression in *H3F3A* mutated glioblastomas

Positive PDGFRA immunohistochemical expression was detected in 30.8% (33/107) of cases. Comparing PDGFRA expression across *BRAF*, *IDH1* and *H3F3A* mutated glioblastomas, positive expression co-occurred in 50% (10/10) of *H3F3A* mutated tumors, 27.8% (5/13) of *IDH1* mutated tumors and 12.5% (2/16) of *BRAF* mutated tumors (*p* = 0.05). Among *H3F3A* mutated tumors, 100% (3/3) of G34V/R mutated tumors and 42.2% (7/17) of K27M mutated tumors demonstrated PDGFRA expression.

### Prognostication of young adult glioblastoma by molecular biomarkers

**Figure 3:**
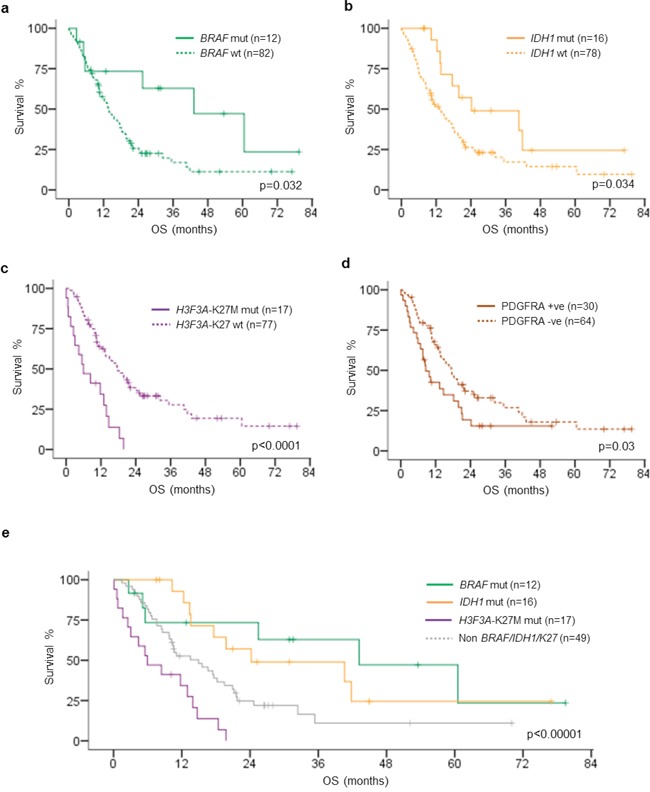
Kaplan–Meier survival analysis of *BRAF* mutation, *IDH1* mutation, *H3F3A*-K27M mutation, PDGFRA immunohistochemistry positivity and subgroups defined by *BRAF*, *IDH1*, *H3F3A*-K27M mutations **a.** *BRAF*-V600E mutation was associated with longer OS comparing to *BRAF* wild type (*p* = 0.032). **b.** *IDH1*-R132H mutation was associated with longer OS comparing to *IDH1* wild type (*p* = 0.034). **c.** *H3F3A*-K27M mutation was associated with shorter OS comparing to *H3F3A* wild type (*p* < 0.0001). **d.** PDGFRA immunohistochemistry positivity was associated with OS comparing to PDGFRA immunohistochemistry negativity (*p* = 0.03). **e.** *BRAF*-V600E mutated tumors exhibited the longest OS followed by *IDH1*-R132H mutated tumors, while *H3F3A*-K27M mutated tumors showed the worst OS (*p* < 0.00001). OS, overall survival; mut, mutated; wt, wild type.

We investigated the survival data of the young adult glioblastoma cohort according to the clinical parameters and molecular biomarkers by univariate analysis as shown in Table 2. Patients receiving total resection (*p* = 0.002) and chemo-radiotherapy (*p* < 0.0001) were associated with better prognosis and midline tumor location was associated with poor outcome (*p* < 0.0001) (Supplementary Figure S2). Among the molecular biomarkers evaluated, *BRAF*, *H3F3A*-K27M, *IDH1* and PDGFRA demonstrated prognostic relevance in young adult glioblastomas. *BRAF* mutated glioblastomas exhibited better prognosis than those with wild-type *BRAF* (*p* = 0.032). The median overall survival was 43.2 months for *BRAF* mutated glioblastomas and 13.6 months for *BRAF* wild-type glioblastomas. *IDH1* mutated tumors, as expected, also exhibited favorable prognosis comparing to *IDH1* wild-type tumors, with median overall survival of 24.2 months in the mutant group and 13.5 months in the wild-type group (*p* = 0.034). *H3F3A*-K27M mutation was associated with poor prognosis across the cohort. The median overall survival was 6 months in K27M mutated tumors, compared to 17.6 months in the wild-type counterparts (*p* < 0.0001). Tumors with positive PDGFRA expression showed shorter survival (8.6 months) than those with negative expression (17.4 months) (*p* = 0.03). Co-evaluation of *BRAF*, *IDH1* and *H3F3A*-K27M status stratified young adult glioblastomas into four prognostic groups across the cohort (*p* < 0.00001). *BRAF* mutated tumors (*p* = 0.038) and *IDH1* mutated tumors (*p* = 0.028) had better prognosis than *BRAF/IDH1/*K27 wild-type tumors, which in turn showed better survival than K27M mutated tumors (*p* = 0.002) (Figure 3a to 3e). Subset analysis demonstrated that positive PDGFRA expression was associated with poor outcome within the K27M mutated glioblastoma subgroup (*p* = 0.03) (Supplementary Figure S2). Multivariate analysis was performed by including molecular biomarkers showing prognostic relevance in univariate analysis and adjusted for patient age, tumor location, operation and adjuvant treatment. Older patient age (HR = 1.085, 95% CI = 1.015 to 1.159, *p* = 0.016) and tumors involving midline structure (HR = 3.86, 95% CI = 1.509 to 9.877, *p* = 0.005) independent poor prognostic factors. Tumors treated by concomitant chemo-radiotherapy (HR = 0.196, 95% CI = 0.072 to 0.531, *p* = 0.001) and *IDH1* mutation (HR = 0.389, 95% CI = 0.172 to 0.878, *p* = 0.023) were independent favorable prognostic factors.

**Table 2:** Univariate analysis of clinical parameters and molecular markers

	*n*	HR	[95% CI]	Median OS (months)	*p*
Age					
≤ 25 years	54	1		17.6	0.094
> 25 years	53	1.5	[0.93 – 2.4]	12.3	
Tumor location					
Midline	16	3.503	[1.974 – 6.216]	8.4	<0.0001
Non-midline	78	1		18.2	
Operation					
Total resection	58	0.456	[0.271 – 0.767]	19.8	0.002
Non-total resection	29	1		11.1	
Adjuvant therapy					
Chemotherapy + Radiotherapy	52	0.089	[0.036 – 0.221]	19.8	<0.0001
Chemotherapy only	12	0.176	[0.063 – 0.494]	7.6	
Radiotherapy only	9	0.289	[0.098 – 0.848]	10.3	
No adjuvant treatment	7	1		4.5	
*BRAF*					
Mutant	12	0.405	[0.173 – 0.951]	43.2	0.032
Wild-type	82	1		13.6	
*IDH1*					
Mutant	16	0.476	[0.236 – 0.962]	24.2	0.034
Wild-type	78	1		13.5	
*H3F3A*-K27					
Mutant	17	3.448	[1.91 – 6.225]	6	<0.0001
Wild-type	77	1		17.6	
*H3F3A*-G34					
Mutant	3	0.331	[0.046 – 2.389]	NR	0.248
Wild-type	91	1		14.7	
*TERT*p					
Mutant	6	1.492	[0.539 – 4.134]	5.1	0.438
Wild-type	85	1		15.6	
*EGFR*					
Amplified	3	1.521	[0.476 – 4.857]	10.7	0.476
Non-amplified	91	1		15	
*CDKN2A*					
Homozygous deletion	28	0.616	[0.35 – 1.086]	13.9	0.091
No homozygous deletion	47	1		15	
PDGFRA expression					
Positive	30	1.732	[1.048 – 2.862]	8.6	0.03
Negative	64	1		17.4	

HR, hazard ratio; 95% CI, 95% confidence interval

## DISCUSSION

While the predominant middle-aged and elderly glioblastomas as well as the pediatric glioblastomas have been extensively investigated, there is lack of focusing study in the young adult age group. In this study, we showed that prognostication and possibly classification of young adult glioblastoma can be biomarker-based and demonstrated that a significant portion of young adult glioblastomas could be genetically defined by mutually exclusive *BRAF*-V600E mutation (15%), *H3F3A*-K27M mutation (15.9%), *H3F3A*-G34R/V mutation (2.8%) and *IDH1*-R132H mutation (16.8%). *BRAF*-V600E mutation was frequently identified in pediatric low-grade gliomas including pleomorphic xanthoastrocytoma, ganglioglioma and extra-cerebellar pilocytic astrocytoma [14, 15]. In contrast to these pediatric low-grade glioma subtypes, mutation frequency was much lower in adult diffuse gliomas [15–19]. Knobbe and co-workers investigated 94 glioblastomas and identified three cases (3.2%) harboring *BRAF* mutation [16]. In the study by Schindler and colleagues examining 1,320 nervous system tumors, *BRAF* mutation was detected in less than 2% of adult glioblastomas investigated [15]. Dahiya and colleagues evaluated the *BRAF* status in 39 adult glioblastomas and identified 7.7% (three cases) possessing the mutation [19]. The mutation was reported in up to 54% of epithelioid glioblastoma, an uncommon histologic variant of glioblastoma in young adults [17]. In our current study focusing on young adult patients aged from 17 to 35 years, 15% of patients had *BRAF* mutation but no *BRAF* mutation was identified in seven cases of secondary young adult glioblastomas. And patients had *BRAF* mutation were significantly younger than those without the mutation. Mutated cases had mean age of 22.3 years and median age of 21 years, compared to mean age of 25.5 years and median age of 26 years in wild-type cases (*p* = 0.013). Additionally, *BRAF* mutation identified a subset of patients with favorable prognosis in our cohort. In univariate analysis, the median overall survival of patients with *BRAF* mutated glioblastomas was 43.2 months and those with wild-type *BRAF* was 13.6 months (*p* = 0.032). Notably, among the 16 *BRAF* mutated glioblastomas, 11 cases had operation record and total resection could be achieved in all cases (*p* = 0.013), suggesting *BRAF* mutated glioblastomas were more amendable to surgical resection. Seven cases received concomitant chemotherapy and radiotherapy, out of nine cases with adjuvant treatment data available. Since majority of the *BRAF* mutated tumors received total resection and concomitant chemo-radiation which could account for the favorable outcome of this subset of patient in our cohort, we tried to analyze the prognostic value of *BRAF* mutation in patients receiving total resection and concomitant chemoradiation (*n* = 37). Seven *BRAF* mutated tumors showed strong trend of better prognosis than 30 *BRAF* wild-type tumors (Supplementary Figure S2). The potential prognostic value of *BRAF*-V600E mutation was also recently reported in pediatric high-grade gliomas [9, 12]. Mistry and colleagues described a series of pediatric secondary high-grade gliomas harboring *BRAF* mutation and *CDKN2A* deletion, showing longer latency to transformation from low-grade lesion and better clinical outcome [12]. By conducting genome-wide DNA methylation profiling in 202 pediatric glioblastomas, Korshunov and colleagues identified an epigenomic subset of glioblastoma showing methylation pattern similar to pleomorphic xanthoastrocytoma, enriched for *BRAF* mutation and *CDKN2A* homozygous deletion, and showed favorable prognosis [9]. Although those studies were on pediatric glioblastomas, our study provided complementary results to theirs and supported the clinical significance of *BRAF*-V600E testing in glioblastoma of young person [9, 12, 17, 19]. Apart from the potential prognostic value, *BRAF*-V600E mutation also served as a novel therapeutic target in this subset of glioblastomas. Robinson and colleagues recently reported a case of *BRAF* mutated pediatric glioblastoma treated by *BRAF* inhibitor vemurafenib and showed complete response [20]. Given the potential clinical utility in prognostication and treatment selection, *BRAF* mutational testing, either by direct sequencing or immunohistochemistry [21], should be conducted in glioblastomas of young patients.

Histone H3 mutation was exclusively observed in *H3F3A* in 18.7% of young adult glioblastomas. *HIST1H3B* mutation was not found in any of the 107 young adult samples examined, suggesting the mutation was specific to diffuse intrinsic pontine gliomas (DIPG) but not for non-brainstem high grade gliomas in both pediatric and adult patients [7, 22]. K27M and G34R/V mutated tumors showed distinct tumor locations and clinical outcome in our cohort as in pediatric glioblastomas [9, 10]. In our cohort, vast majority of K27M mutated tumors were located in midline structures including thalamus, ventricular system and cervical/thoracic spinal cord. These rare midline glioblastomas, despite of their different anatomical locations, shared the same driver mutation and suggested a closely-related origin [10, 23]. Notably, K27M mutated glioblastomas showed aggressive clinical course in our cohort with median overall survival of 6 months (range 0.1 months to 19.8 months). Within this aggressive subset of glioblastoma, tumors with positive PDGFRA immunohistochemical expression exhibited a significantly shorter survival (median 2.5 months) than tumors with negative expression (median 11.7 months) (*p* = 0.03). Interestingly, Puget and colleagues previously reported a distinct transcriptional subgroup of DIPG characterized by oligodendroglial differentiation, driven by PDGFRA upregulation and exhibited significantly worse outcome [23]. The association of PDGFRA expression with poor prognosis in K27M mutated glioblastomas in our study was in line with previous observation. The prognostic value of PDGFRA expression/alterations in the K27M mutated tumors warranted further evaluation in a larger cohort.

*IDH1* mutation was present in 16.8% of young adult glioblastomas of which the mutation frequency was comparatively higher than the predominant group of adult/elderly glioblastoma as well as pediatric glioblastomas [8, 9, 24–27]. Previous study by Pollack and colleagues reported that *IDH1* mutation was common in adolescent malignant gliomas in which 16.3% of high grade gliomas between 3 to 21 years harbored the mutation and was significantly associated with age greater than 14 years [28]. As expected, *IDH1* mutated glioblastomas showed favorable prognosis in our cohort which was independent of age, tumor location, operation and adjuvant treatment among the young adult patients. Favorable prognostic value of *IDH1* mutation was also recently demonstrated in pediatric glioblastomas [9]. Notably, although *IDH1* mutation was identified in nearly 90% of secondary elderly glioblastomas [27], only 43% (3/7) of *IDH1* mutation was detected in our young adult glioblastomas, suggesting that *IDH1* mutation wasn't a main contributing marker for young secondary glioblastomas. Collectively, our study provided complementary evidence to previous studies that *IDH1* mutated glioblastoma was a glioblastoma subgroup with favorable prognosis prevalent in adolescents and young adults [11].

Hotspot mutations in *TERT* promoter region were identified in up to 80% of adult/elderly glioblastomas [29–31] but rare in pediatric glioblastomas [32]. The prevalence of *TERT*p mutation in the young adult age group was not precisely reported. In our study, only 8.7% of young adult glioblastomas harbored *TERT*p mutation, suggesting that mutation induced telomerase activation might not be a major mechanism in telomere deregulation in that age group. It remained to be investigated if promoter methylation of *TERT* causing telomerase upregulation or the alternative lengthening of telomeres (ALT) represented the major mechanism of telomere maintenance in young adult glioblastomas [6, 33]. *TERT*p mutations were associated with poor prognosis in glioblastomas [30, 31, 34, 35] as well as lower-grade gliomas with wild-type *IDH* [34, 36, 37]. Although not reached statistical significance, the median overall survival of patients with *TERT*p mutated glioblastomas was only 5.1 months, compared to 15.6 months in those with wild-type *TERT*p. Notably, three *TERT*p mutations were overlapped with *BRAF*-V600E mutation in the cohort and overall survival was 5.1 months in one patient with survival data available. *BRAF*-V600E and *TERT*p mutations were recently found cooperatively identifying the most aggressive subset of papillary thyroid cancer with high recurrence rate [38]. *TERT*p mutations, either C228T or C250T, generated a consensus binding site (5′-TTCC-3′) for E-twenty-six (ETS) transcription factors and upregulated *TERT* expression [34, 39, 40]. On the other hand, activation of mitogen-activated protein kinase pathway was also shown to cause upregulation of the ETS system [41–43]. The synergistic effect of *BRAF*-V600E and *TERT*p mutations in promoting tumorigenesis was therefore biologically explainable [38]. Although only 2.9% (3/104) of patients harbored concurrent *BRAF*-V600E and *TERT*p mutation, this subgroup accounted for 18.8% (3/16) of *BRAF* mutated glioblastomas of young adults. Further study should be conducted to evaluate the clinical value of concurrent *BRAF* and *TERT*p mutations in young adult glioblastomas.

In summary, our study demonstrates recurrent *BRAF*, *IDH1* and *H3F3A* mutations in young adult glioblastomas with clinical impacts. *BRAF* mutation and *IDH1* mutation identify glioblastomas with less aggressive clinical course and *H3F3A*-K27M mutation defines glioblastomas with dismal prognosis. The biomarker-based stratification has clinical implications and refines the prognostic classification of young adult glioblastomas.

## MATERIALS AND METHODS

### Patients and tissue samples

A total of 107 tissue samples (all formalin-fixed paraffin-embedded) were obtained from young adult patients (age from 17–35 years) of Department of Anatomical and Cellular Pathology, Prince of Wales Hospital (Hong Kong) and Department of Neurosurgery, Huashan hospital (Shanghai) with a histological diagnosis of “glioblastoma, WHO grade IV”. Seven out of the 107 samples were secondary glioblastomas progressed from low grade gliomas, including oligoastrocytoma and astrocytoma. Other 102 cases were diagnosed as primary glioblastomas. Clinical and survival data of the patients were retrieved from the respective institutional medical record systems. This study was approved by the Ethics Committee of Shanghai Huashan Hospital and the New Territories East Cluster-Chinese University of Hong Kong Ethics Committee.

### Molecular analysis

Mutational analysis was performed as described previously [36, 44]. Tissues from representative tumor area with tumor content 70% were scrapped off from dewaxed sections and treated with proteinase K at a final concentration of 2 mg/ml in 10 mM Tris-HCl buffer (pH 8.5) at 55°C for 2–18 hours and then at 98°C for 10 minutes. Crude cell lysate was centrifuged and supernatant was used for subsequent PCR analysis. The forward and reverse primers then were used to amplify gene *BRAF*, *H3F3A*, *HIST1H3B*, *IDH1* and *TERT*. PCR was performed in 10ul reaction mixture for different thermal protocol (Supplementary Table S1, S2). Sequencing was performed using BigDye Terminator Cycle Sequencing kit v1.1 (Life Technologies). The products were resolved in Genetic Analyzer 3130 × l and analyzed by Sequencing Analysis software. Hotspots *BRAF*-V600E, *H3F3A*-K27M, *H3F3A*-G34R/V, *HIST1H3B*-K27M, *IDH1*-R132H and *TERT*p were detected (Supplementary Figure S1). All base changes were confirmed by sequencing of a newly amplified fragment.

### Fluorescence *in situ* hybridization analysis for *EGFR* amplification and *CDKN2A* deletion

Dual-probe fluorescence *in situ* hybridization (FISH) assay was performed on paraffin-embedded sections, with locus-specific probes for *EGFR* and *CDKN2A* paired with centromere probes for chromosome 7p12 and chromosome 9p21 (Supplementary Figure S1, Table S4). Deparaffinization of the sections was carried out, followed by dehydration in 100% ethanol, retrieval by 1M sodium thiocyanate at 80°C for 10 minutes, and digestion in 0.04% pepsin at 37°C was applied on each section for 10 minutes. Simultaneous probe per specimen was denatured at 80°C for 10 minutes with subsequent overnight incubation at 37°C. The sections were washed next day in 1.5 M Urea/2X saline sodium citrate at 50°C for 10 minutes twice. After washing, sections were stained with Vectashield mounting medium containing 4′,6-diamidino-2-phenylindole (Vector Laboratories) and visualized under a fluorescent microscope (Carl Zeiss Microscopy LLC, NY, USA). Hybridizing signals in at least 100 non-overlapping nuclei were counted. *EGFR* amplification was considered when 50% of the tumor cells harbored more than five signals per nuclei CEP7 or innumerable tight clusters of signals of the locus probe [45]. *CDKN2A* deletion was considered if both signals were lost (homozygous deletion) in at least 20% of tumor nuclei [46].

### Immunohistochemistry of PDGFRA

FFPE tissue sections of 4 micron thickness were deparaffinized in xylene and rehydrated in graded alcohols. For PDGFRA, antigen retrieval was carried out by treating the sections in 1M Citrate buffer (PH = 6.0) in a microwave oven. After antigen retrieval, all sections were processed by BondMax automade staining systems (Leica BondMax) using validated protocols. Tissue sections were incubated at 37°C for 30 mins with relevant antibodies of PDGFRA (Supplementary Table S3). Antigen detection was performed using Ultra View diamino benzidine chromogen step (BondMax). The presence of cytoplasmic and membrane staining indicated positivity for PDGFRA [47] (Supplementary Figure S1, Table S5).

### Statistical analysis

Statistical analysis was performed using IBM SPSS Statistics 20 (IBM Corporation, NY, USA). Correlation between molecular markers and clinical parameters were examined by *X*^2^-test. Comparison between two groups was performed by Student's *t*-test or Mann–Whitney *U*-test. Comparison between three or more groups used one-way analysis of variance (ANOVA). Overall survival (OS) was defined as the duration between the diagnosis and death or last follow-up [25]. Survival curves were plotted by Kaplan-Meier method and analyzed by Log-rank test. Multivariate analysis for independent prognostic marker was performed by Cox-proportional hazards model. Tests with a *p* value below 0.05 were considered significant.

Supplementary information is available at *Oncotarget*'s website.

## SUPPLEMENTARY FIGURES AND TABLES


