# Supplementary material for: Biomarker-based prognostic stratification of young adult glioblastoma
Source: Oncotarget. 2015 Oct 5;7(4):5030–41. doi: 10.18632/oncotarget.5456 (PMC4826263; doi:10.18632/oncotarget.5456)
Supplement: Supplementary file 1 [file oncotarget-07-5030-s001.pdf]

# SUPPLEMENTARY FIGURES AND TABLES

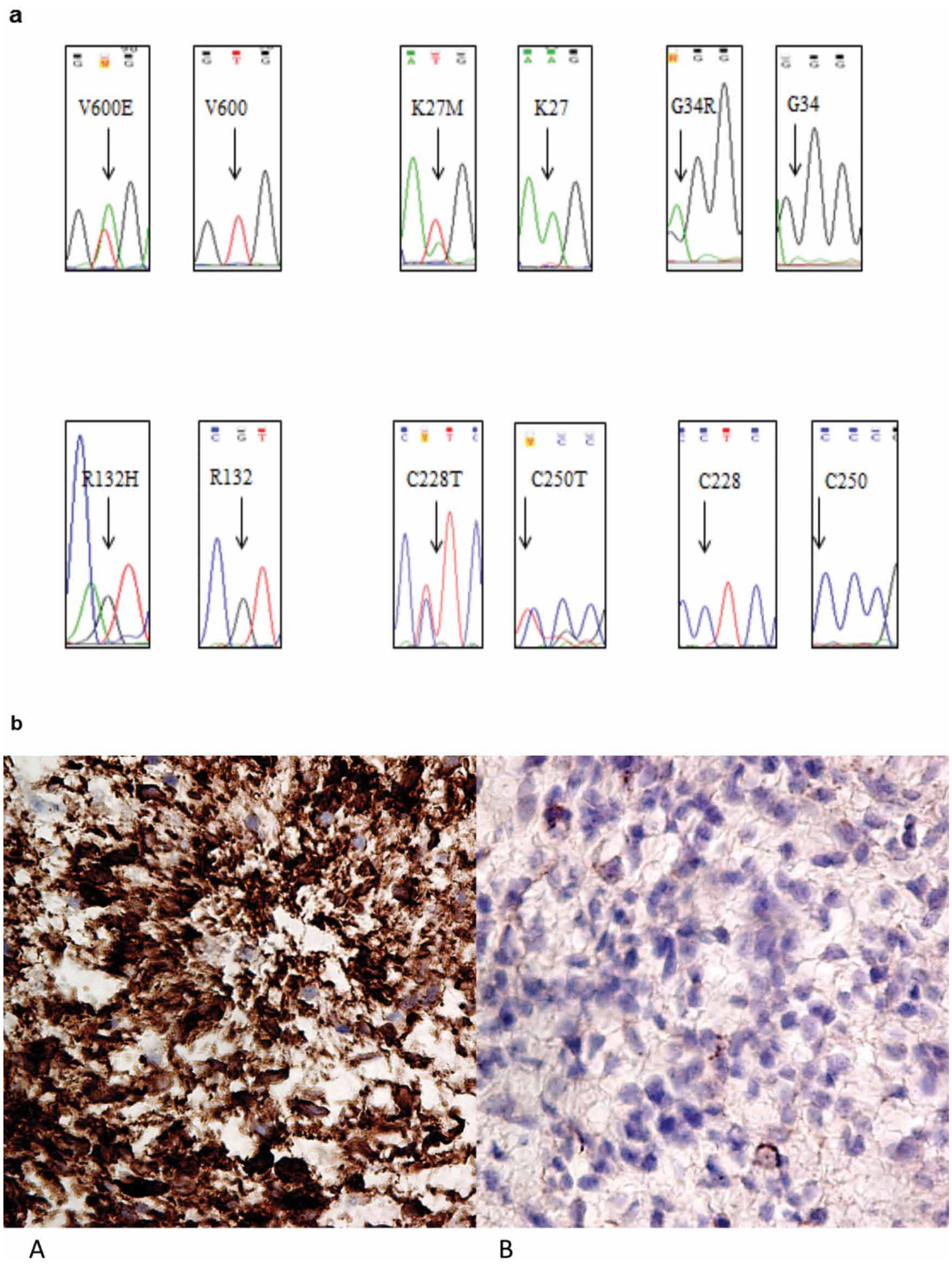


**Supplementary Figure S1: a.** Hotspot wild types and mutations of BRAF-V600E, H3F3A-K27M, H3F3A-G34R, IDH1-R132H and TERTp-C228T, TERTp-C250T. **b.** Representative photograph of PDGFRA immunohistochemistry × 400 original magnification. (A) Cytoplasmic and membrane immunohistochemistry positivity of PDGFRA. (B) Cytoplasmic and membrane immunohistochemistry negativity of PDGFRA. (*Continued*)

c

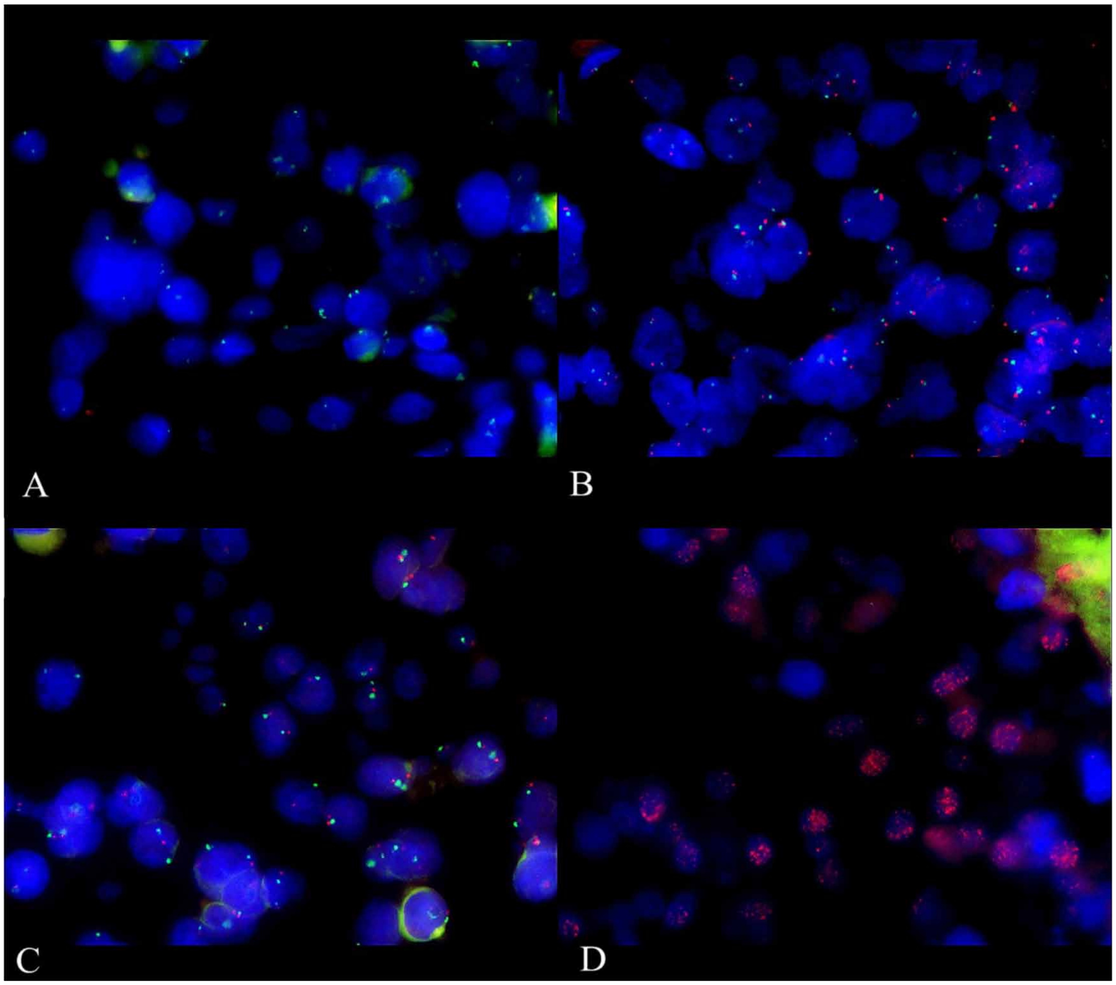


**Supplementary Figure S1: (*Continued*) c.** Representative photograph of *CDKN2A* and *EGFR* fluorescence *in situ* hybridization (FISH). (A) *CDKN2A* homozygous deletion. Loss of both locus-specific signals with presence of control signals. (B) *CDKN2A* Intact. Two test signals and two control signals. (C) *EGFR* Intact. Two test signals and two control signals. (D) *EGFR* amplification (cluster). Numberless locus-specific signals.

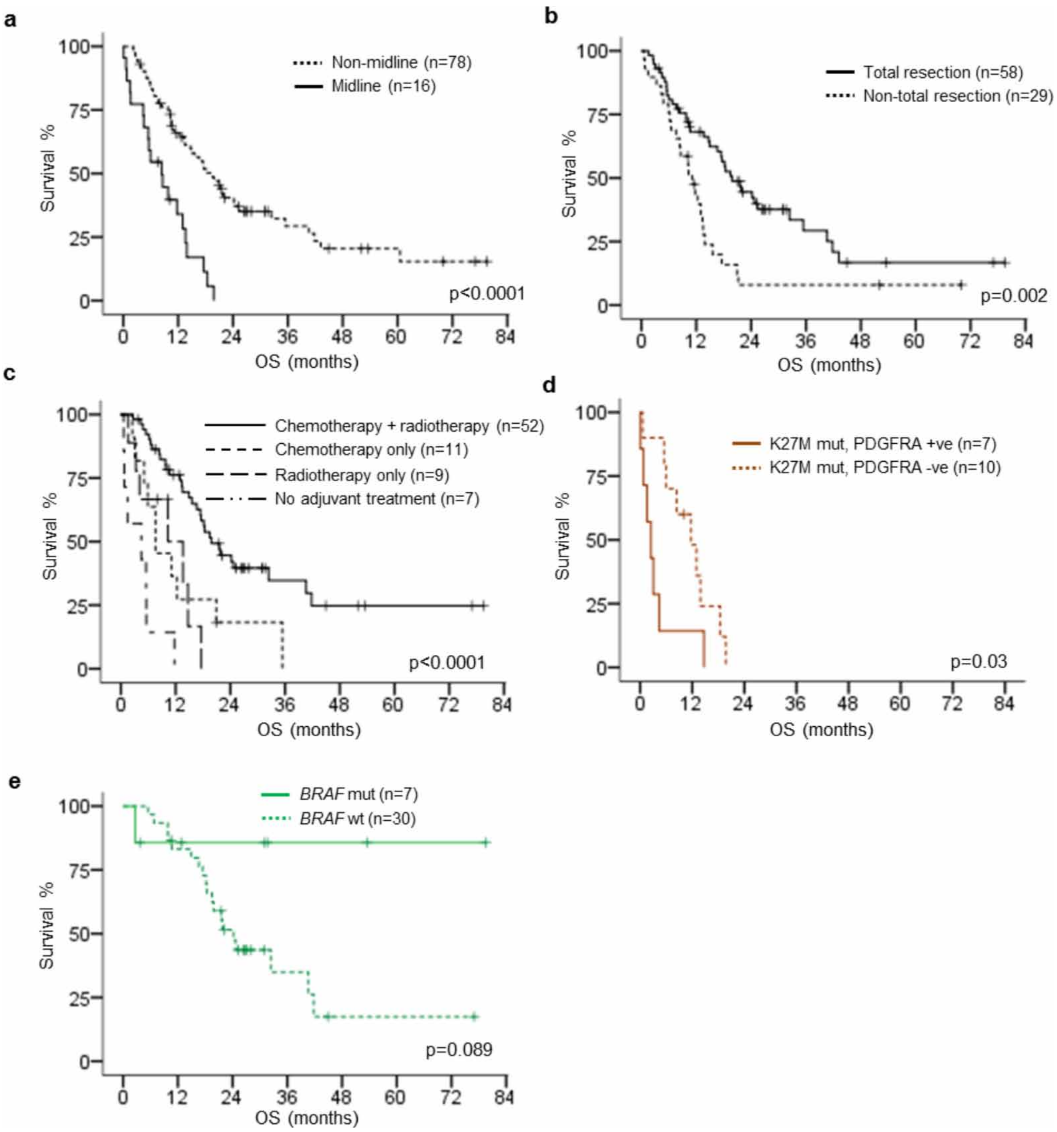


**Supplementary Figure S2: Kaplan–Meier survival analysis of tumor location, treatment methods, H3F3A-K27M mutation and PDGFRA immunohistochemistry positivity. a.** Midline tumor location was associated with shorter OS comparing to non-midline tumor location ($p < 0.0001$). **b.** Patients who accepted total tumor resection survive better than those with non-total resection ($p = 0.002$). **c.** Patients who received both chemotherapy and radiotherapy survive better than those with only one of these adjuvant treatment, and patients who received no adjuvant treatment showed the shortest OS ($p < 0.0001$). **d.** Within *H3F3A*-K27M mutated tumors, PDGFRA immunohistochemistry positivity was associated with shorter OS ($p = 0.03$). **e.** Within patients who received total resection and concomitant chemoradiation, *BRAF* mutated tumors showed strong trend of better prognosis than *BRAF* wild-type tumors ($p = 0.089$). OS, overall survival.

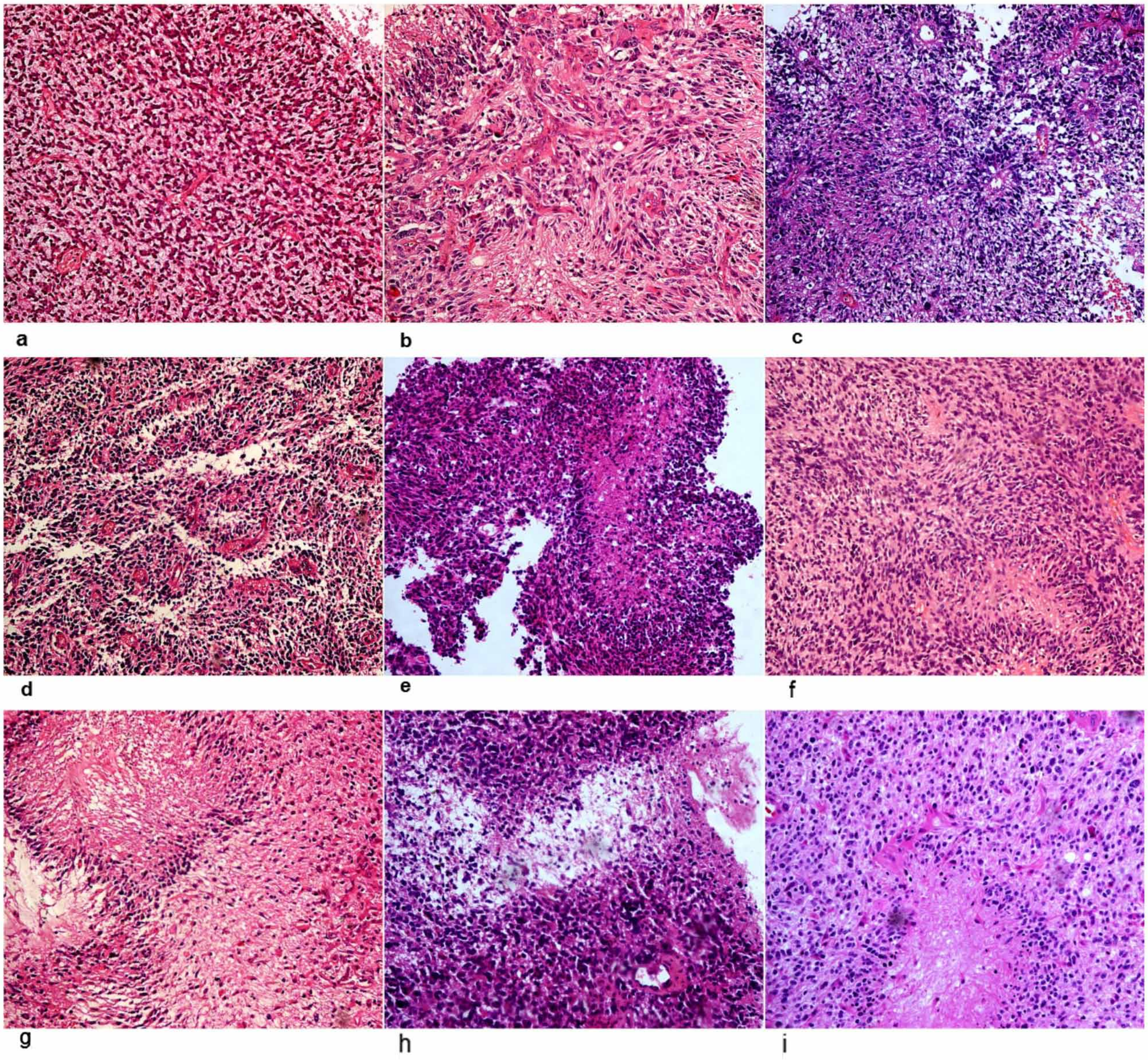

**Supplementary Figure S3: H&E photographs of representative H3F3A, BRAF and IDH1 mutated cases. a, b, c.** Representative H&E photographs of H3F3A mutated cases. **d, e, f.** Representative H&E photographs of BRAF mutated cases. **g, h, i.** Representative H&E photographs of IDH1 mutated cases.

## Supplementary Table S1: Primers used for gene amplification in this study

| Gene | Forward | Reverse | Expected Size |
|---|---|---|---|
| H3F3A | 5′-CATGGCTCGTACAAAGCAGA-3′ | 5′-CAAGAGAGACTTTGTCCCATTTTT-3′ | 170bp |
| HIST1H3B | 5′-TTTCCTTTCCTCCACAGACG-3′ | 5′-CGGTAACGGTGAGGCTTTT-3′ | 180bp |
| BRAF | 5′-TGCTTGCTCTGATAGGAAAATG-3′ | 5′-CCACAAAATGGATCCAGACA-3′ | 173bp |
| IDH1 | 5′-CGGTCTTCAGAGAAGCCATT-3′ | 5′-CACATTATTGCCAACATGAC-3′ | 122bp |
| TERT | 5′-GTCCTGCCCCTTCACCTT-3′ | 5′-CAGCGCTGCCTGAAACTC-3′ | 163bp |

**Supplementary Table S2: PCR kit and thermal cycling protocol for *BRAF*, *IDH1*, *H3F3A*, *HIST1H3B* and *TERT***

| **BRAF PCR cycling protocol:** | | | |
|---|---|---|---|
| **PCR Kit:** | **KAPA 2G Robust Hot Start Ready Mix** | | |
| **Step** | **Temperature** | **Duration** | **Cycle** |
| Initial denaturation: | 95°C | 3 mins | 1 |
| Denaturation: | 95°C | 15 secs | 40 |
| Annealing: | 60°C | 20 secs | 40 |
| Extension: | 72°C | 15 secs | 40 |
| Final extension: | 72°C | 1 min | 1 |
| **IDH1 PCR cycling protocol:** | | | |
| **PCR Kit:** | **KAPA 2G Robust Hot Start Ready Mix** | | |
| **Step** | **Temperature** | **Duration** | **Cycle** |
| Initial denaturation: | 95°C | 3 mins | 1 |
| Denaturation: | 95°C | 15 secs | 45 |
| Annealing: | 60°C | 20 secs | 45 |
| Extension: | 72°C | 30 secs | 45 |
| Final extension: | 72°C | 5 mins | 1 |
| **H3F3A PCR cycling protocol:** | | | |
| **PCR Kit:** | **KAPA Hifi Hot Start Ready Mix** | | |
| **Step** | **Temperature** | **Duration** | **Cycle** |
| Initial denaturation: | 95°C | 5 mins | 1 |
| Denaturation: | 98°C | 20 secs | 45 |
| Annealing: | 60°C | 20 secs | 45 |
| Extension: | 72°C | 30 secs | 45 |
| Final extension: | 72°C | 1 min | 1 |
| **HIST1H3B PCR cycling protocol:** | | | |
| **PCR Kit:** | **KAPA Hifi Hot Start Ready Mix** | | |
| **Step** | **Temperature** | **Duration** | **Cycle** |
| Initial denaturation: | 95°C | 5 mins | 1 |
| Denaturation: | 98°C | 20 secs | 45 |
| Annealing: | 64°C | 35 secs | 45 |
| Extension: | 72°C | 30 secs | 45 |
| Final extension: | 72°C | 1 min | 1 |

(*Continued*)

| TERT PCR cycling protocol: | | | |
|---|---|---|---|
| **PCR Kit:** | **KAPA Hifi Hot Start Ready Mix** | | |
| **Step** | **Temperature** | **Duration** | **Cycle** |
| Initial denaturation: | 95°C | 5 mins | 1 |
| Denaturation: | 98°C | 20 secs | 45 |
| Annealing: | 66°C | 15 secs | 45 |
| Extension: | 72°C | 30 secs | 45 |
| Final extension: | 72°C | 1 min | 1 |

## Supplementary Table S3: Antibodies used for immunohistochemistry of PDGFRA

| Antibody | Company | Clone | Isotype | Dilution |
|---|---|---|---|---|
| PDGFRA | Santa Cruz (C-20) | Polyclone C20 Rabbit anti-human | IgG | 1/200 |

## Supplementary Table S4: Probes used for FISH of *EGFR* (7p12) and *CDKN2A* (9p21)

| Gene/Locus | Clone | Source | Fluorescent label |
|---|---|---|---|
| EGFR(7p12) | CTD-2199A14 | Vysis Abbott, USA | FITC |
| CDKN2A(9p21) | Commercial | Vysis Abbott, USA | FITC |

## Supplementary Table S5: All sections were scored by observers blinded to the clinical information and result of the molecular tests

| IHC score | Assessment of PDGFRA IHC staining |
|---|---|
| 0 | No staining in all tumor cells |
| 1+ | Weak cytoplasmic and membrane staining |
| 2+ | Moderate cytoplasmic and membrane staining in > 50% of tumor cells. |
| 3+ | Strong cytoplasmic and membrane staining in > 50% of tumor cells. |

Samples with 0 and 1+ were considered negative expression, and those with 2+ and 3+ were considered positive expression.
